# The sialidase inhibitor 2,3-dehydro-2-deoxy-*N*-acetylneuraminic acid is a glucose-dependent potentiator of insulin secretion

**DOI:** 10.1038/s41598-020-62203-8

**Published:** 2020-03-23

**Authors:** Akira Minami, Yuka Fujita, Sumika Shimba, Mako Shiratori, Yukiko K. Kaneko, Toshiaki Sawatani, Tadamune Otsubo, Kiyoshi Ikeda, Hiroaki Kanazawa, Yasuyo Mikami, Risa Sekita, Yuuki Kurebayashi, Tadanobu Takahashi, Taeko Miyagi, Tomohisa Ishikawa, Takashi Suzuki

**Affiliations:** 1 Department of Biochemistry, School of Pharmaceutical Sciences, University of Shizuoka, Shizuoka, 422-8526 Japan; 2 Department of Pharmacology, School of Pharmaceutical Sciences, University of Shizuoka, Shizuoka, 422-8526 Japan; 3 Department of Organic Chemistry, School of Pharmaceutical Sciences, Hiroshima International University, Hiroshima, 737-0112 Japan; 4 Department of Functional Anatomy, School of Nursing, University of Shizuoka, Shizuoka, 422-8526 Japan; 5 Miyagi Cancer Center Research Institute, Natori, 981-1293 Japan

**Keywords:** Glycoconjugates, Glycoconjugates, Glycobiology, Glycobiology

## Abstract

Sialidase cleaves sialic acid residues from a sialoglycoconjugate: oligosaccharides, glycolipids and glycoproteins that contain sialic acid. Histochemical imaging of the mouse pancreas using a benzothiazolylphenol-based sialic acid derivative (BTP3-Neu5Ac), a highly sensitive histochemical imaging probe used to assess sialidase activity, showed that pancreatic islets have intense sialidase activity. The sialidase inhibitor 2,3-dehydro-2-deoxy-*N*-acetylneuraminic acid (DANA) remarkably enhances glutamate release from hippocampal neurons. Since there are many similar processes between synaptic vesicle exocytosis and secretory granule exocytosis, we investigated the effect of DANA on insulin release from β-cells. Insulin release was induced in INS-1D cells by treatment with 8.3 mM glucose, and the release was enhanced by treatment with DANA. In a mouse intraperitoneal glucose tolerance test, the increase in serum insulin levels was enhanced by intravenous injection with DANA. However, under fasting conditions, insulin release was not enhanced by treatment with DANA. Calcium oscillations induced by 8.3 mM glucose treatment of INS-1D cells were not affected by DANA. Blood insulin levels in sialidase isozyme Neu3-deficient mice were significantly higher than those in WT mice under *ad libitum* feeding conditions, but the levels were not different under fasting conditions. These results indicate that DANA is a glucose-dependent potentiator of insulin secretion. The sialidase inhibitor may be useful for anti-diabetic treatment with a low risk of hypoglycemia.

## Introduction

Sialidase is a hydrolase that removes sialic acid from a sialoglycoconjugate. Mammalian sialidase has four isozymes: Neu1, Neu2, Neu3 and Neu4. These four isozymes have differences in the tissues where they are expressed, their subcellular locations, their substrate specificity and their pH dependency^1^. It has been proposed that sialidase contributes to the regulation of insulin signalling and sensitivity. Neu1, a lysosomal sialidase, regulates insulin signalling for energy metabolism and glucose uptake^2^. Neu3, a plasma membrane-associated sialidase, is associated with tissue-specific insulin sensitivity and glucose tolerance^3–5^. Transgenic mice overexpressing Neu3 mainly in muscles developed severe insulin-resistant diabetes mellitus associated with hyperinsulinemia, islet hyperplasia and increased β-cell mass^5^. Hepatic Neu3 overexpression, however, improves insulin sensitivity and glucose tolerance and is associated with changes in ganglioside composition and peroxisome proliferator-activated receptor gamma signalling^4^.

Recently, we reported that sialidase downregulates glutamate release from neurons^6^. Since sialidase activity increases rapidly in response to neural excitation, sialidase is thought to play a role in the negative-feedback mechanism for glutamate release^7^. In an effort to understand how sialidase might regulate synaptic transmission, we found that the sialidase inhibitor 2,3-dehydro-2-deoxy-*N*-acetylneuraminic acid (DANA) enhanced synaptic vesicle exocytosis in hippocampal neurons^6^. Although there are some differences between synaptic vesicle exocytosis and secretory granule exocytosis, the two have many common processes, including docking, fusion and the recruitment of vesicles at the plasma membrane^8^. DANA may thus have an influence on insulin release as well; however, the role of sialidase in insulin secretion remains unknown.

BTP3-Neu5Ac, a benzothiazolyl-phenol-based sialic acid derivative, is a highly sensitive fluorescent probe used for histochemical imaging of sialidase activity^9^. In the course of staining with BTP3-Neu5Ac in several mammalian tissues, we found that pancreatic islets show intense sialidase activity in the mouse pancreas. On the basis of this observation, we investigated the effect of the sialidase inhibitor DANA on insulin secretion in this study. We show here that DANA enhanced the insulin release induced by a high concentration of glucose, resulting in a decrease in blood glucose levels. Enhancement of insulin release by DANA, however, was not induced under fasting conditions. These results suggested that DANA has intense insulinotropic activity that is glucose-dependent.

## Results

### Distribution of sialidase activity in the mouse pancreas

**Figure 1:**
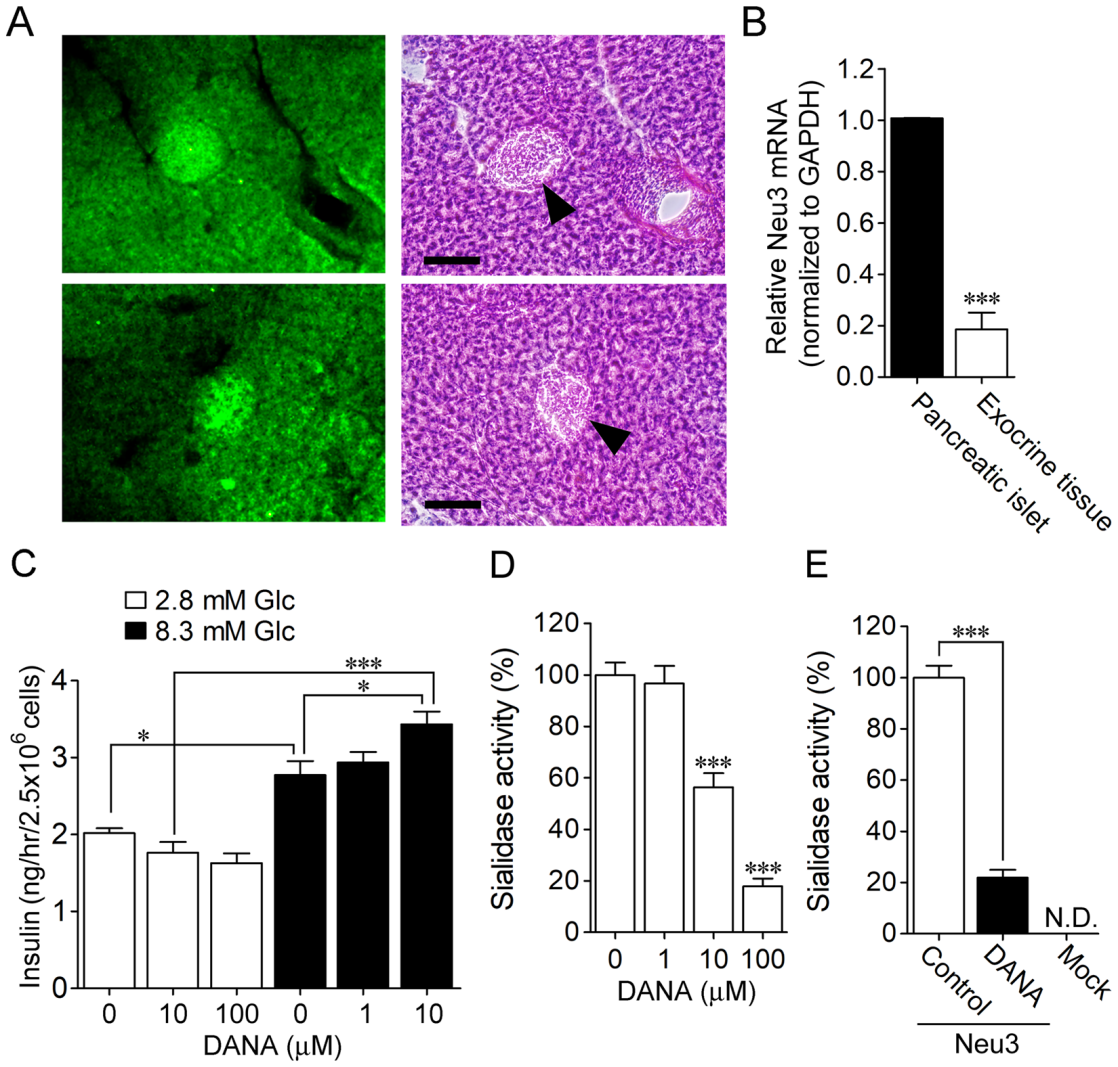
Glucose-dependent insulinotropic activity of the sialidase inhibitor DANA. (**A**) Tails of mouse pancreases were stained with 100 µM BTP3-Neu5Ac (left column) and haematoxylin-eosin (right column). Black arrowheads, pancreatic islets. Scale bars, 0.1 mm. (**B**) Expression levels of Neu3 mRNA in pancreatic islets and exocrine tissues were measured by real-time RT-PCR. n = 4 in each. ***P < 0.001 (unpaired t-test). (**C**) The effect of DANA on insulin secretion in INS-1D cells was investigated with or without 8.3 mM glucose stimulation. *n* = 5–7. (**D**,**E**) The effect of DANA on the sialidase activity of INS-1D cells (**D**) and rat Neu3 (**E**) was investigated using 4MU-Neu5Ac. *P < 0.05 and ***P < 0.001 (one-way ANOVA with Bonferroni’s multiple comparison test). *P < 0.05 and ***P < 0.001 (one-way ANOVA with Bonferroni’s multiple comparison test).

To investigate the distribution of sialidase activity in the pancreas, mouse pancreas sections (100 μm in thickness) were stained with 1 mM BTP3-Neu5Ac at pH 7.3 for 60 min. The pancreatic islets identified by staining with haematoxylin-eosin showed sialidase activity that was intense compared to that observed in exocrine tissues (Fig. 1A).

Sialidase isozyme Neu3 is involved in insulin signalling and insulin-resistant diabetes mellitus^3–5^. The expression level of Neu3 mRNA in mouse pancreatic islets was remarkably higher than that of exocrine tissue (Fig. 1B).

### Insulinotropic effect of DANA

Although pancreatic islets showed intense sialidase activity, the role of sialidase in pancreatic islets is poorly understood. In this study, we investigated the effect of a sialidase inhibitor on insulin secretion in INS-1D clonal β-like cells. Glucose was used at a concentration of 8.3 mM, which is a concentration that is known to cause moderate insulin secretion. Insulin secretion induced by 8.3 mM glucose was enhanced by the addition of 10 μM DANA (Fig. 1C). Hypoglycaemia is the most common and serious side effect of glucose-lowering drugs. Here, we investigated the effect of DANA on insulin release under non-stimulated conditions. Not only 10 μM but also 100 μM DANA did not cause insulin release from INS-1D cells treated with 2.8 mM glucose (Fig. 1C). To evaluate the inhibitory effect of DANA on sialidase in INS-1D cells, sialidase activity of INS-1D cells was measured using 4-methylumbelliferyl-*α*-d-*N*-acetylneuraminic acid (4MU-Neu5Ac), which is a fluorescent substrate used for sialidase activity quantification. Sialidase activity in INS-1D cells was significantly inhibited by treatment with 10–100 μM DANA (Fig. 1D). We also confirmed that Neu3 activity was significantly inhibited by 10 μM DANA (Fig. 1E).

**Figure 2:**
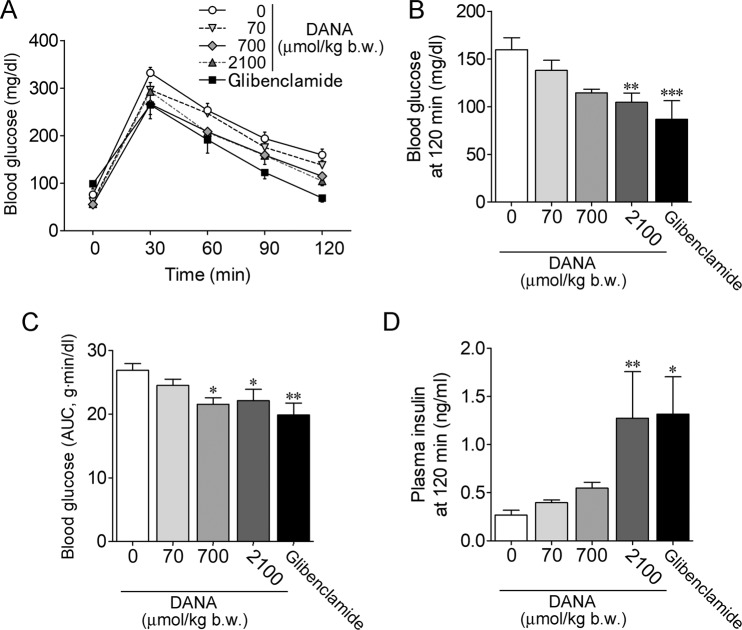
Insulinotropic effect of the sialidase inhibitor DANA. (**A**) An IPGTT was performed after intravenous injection of DANA (70–2,100 μmol/kg b.w.) and intraperitoneal injection of glibenclamide (10 mg/kg b.w.). Glucose (2 g/kg b.w.) was administered intraperitoneally at 0 min. (**B**,**C**) Blood glucose levels at 120 min are indicated by AUC, as shown in (**B,C**). (**D**) Plasma insulin level at 120 min. Control (0 μmol/kg b.w. DANA, *n* = 15–16). Others (*n* = 6–8). *P < 0.05, **P < 0.01 and ***P < 0.001 vs. control (one-way ANOVA with Dunnett’s multiple comparison test).

Next, we investigated the effect of DANA on blood glucose and plasma insulin levels during an intraperitoneal glucose tolerance test (IPGTT) in mice. Glibenclamide is a popular sulfonylurea drug that potently increases insulin secretion by inhibiting ATP-sensitive K^+^ channels independent of blood glucose levels^10^, and it was used as a positive control in the IPGTT. The blood glucose level at 120 min after intraperitoneal injection with glucose (2 g/kg b.w.) was attenuated by DANA (70–2,100 μmol/kg b.w., intravenous injection) in a concentration-dependent manner, and it was attenuated by a high dose of glibenclamide (10 mg/kg b.w., intraperitoneal injection) (Fig. 2A,B). The area under the blood glucose concentration-time curve was significantly decreased by DANA (700 and 2,100 μmol/kg b.w.) and glibenclamide (Fig. 2C). The plasma insulin level at 120 min after glucose injection was increased by treatment with DANA (2,100 μmol/kg b.w.) and glibenclamide (Fig. 2D).

### Effect of DANA on plasma insulin levels under fasting conditions

**Figure 3:**
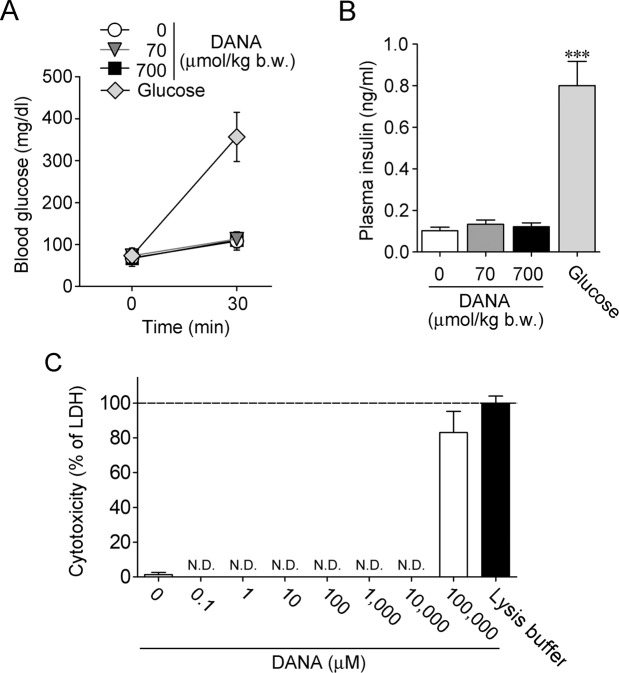
Effect of DANA on insulin release under fasting conditions. (**A**) After 24 hours of fasting, blood glucose levels were measured, and then they were measured again at 30 min after administration of DANA (70 or 700 μmol/kg b.w.) or glucose (2 g/kg b.w.). (**B**) Plasma insulin levels were measured 30 min after administration of DANA or glucose. *n* = 9–11. ***P < 0.001 vs. control (one-way ANOVA with Dunnett’s multiple comparison test). (**C**) The cytotoxicity of DANA (10^−1^–10^5^ μM) was estimated by LDH release from INS-1D cells. LDH release is shown relative to complete LDH release (100%) by treatment with lysis buffer. N.D.: not detected. *n* = 3–4.

Since insulin secretion was not enhanced by DANA under non-stimulated conditions, we investigated the effect of DANA on blood glucose and plasma insulin levels under fasting conditions. After 24 hours of fasting, mice were given an intravenous injection of DANA (70 or 700 μmol/kg b.w.) or an intraperitoneal injection of glucose (2 g/kg b.w.). Plasma levels of insulin and glucose were not affected by DANA administration, but they were increased by glucose administration (Fig. 3A,B). The cytotoxicity of DANA was estimated by lactate dehydrogenase (LDH) release from INS-1D cells, and cytotoxicity was not detected at less than 10 mM DANA treatment (Fig. 3C).

**Figure 4:**
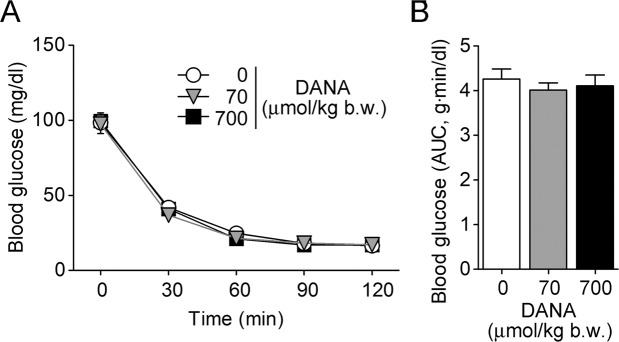
Effect of DANA on insulin sensitivity. (**A**,**B**) After 5 hours of fasting, an insulin tolerance test was performed after intravenous injection of DANA (70 or 700 μmol/kg b.w.). Insulin (2 mU/g b.w.) was administered intraperitoneally at 0 min. The AUC of blood glucose levels is shown in B. *n* = 9 for each.

We also investigated the effect of DANA on insulin sensitivity by an insulin tolerance test. After 5 hours of fasting, mice were given an intravenous injection of DANA just before an intraperitoneal injection of insulin. Blood glucose levels were decreased by insulin but were not affected by DANA (70 or 700 μmol/kg b.w.) (Fig. 4A,B).

### Effect of DANA on [Ca^2+^]_i_ oscillations

**Figure 5:**
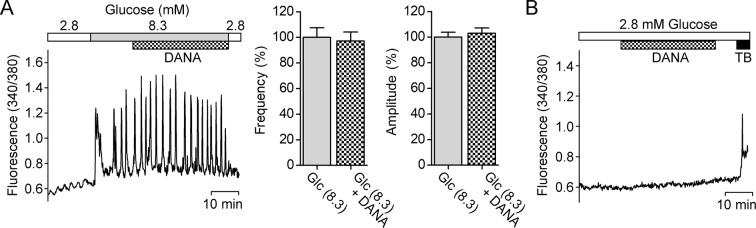
Effect of DANA on [Ca^2+^]_i_ oscillations in β-cells. (**A**) Glucose (8.3 mM) was administered to INS-1D cells to induce [Ca^2+^]_i_ oscillations, and then 10 μM DANA was added. The frequency and amplitude of [Ca^2+^]_i_ oscillations were measured for 5 min before and during treatment with DANA (n = 39). (**B**) INS-1D cells were treated with 10 μM DANA under non-stimulated conditions (2.8 mM glucose). To identify healthy INS-1D cells, tolbutamide (TB) was applied at the end of the experiment. The traces in A and B are representative of 39 and 199 cells from 3 experiments, respectively.

Since [Ca^2+^]_i_ oscillations of β-cells have been well established as a trigger for insulin secretion^11^, we investigated the effects of DANA on [Ca^2+^]_i_ in INS-1D cells. Slow [Ca^2+^]_i_ oscillations induced by 8.3 mM glucose were not affected by 10 μM DANA with frequency or amplitude (Fig. 5A). Under non-stimulated condition (2.8 mM glucose), [Ca^2+^]_i_ was not affected by DANA, but it was increased by the potassium channel blocker tolbutamide (Fig. 5B).

### Effect of DANA on sialic acid expression

**Figure 6:**
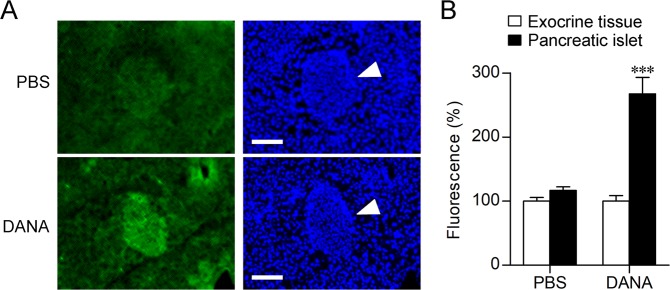
Effect of DANA on sialic acid expression in pancreatic islets. (**A**,**B**) Tails of mouse pancreases were stained with FITC-labelled MAA (green) and DAPI (blue). White arrowheads, pancreatic islets. Scale bars, 0.1 mm. Fluorescence intensities in pancreatic islets and exocrine tissues are shown in B. *n* = 11–12. ***P < 0.001 (two-way ANOVA with Bonferroni’s multiple comparison test).

The effect of DANA on sialic acid expression was investigated by lectin staining. After intravenous injection of DANA or saline, glucose was administered by intraperitoneal injection. At 30 min after glucose administration, sialic acids were detected with *Maackia amurensis* agglutinin (MAA). MAA is a sialic acid-binding lectin that mainly recognizes α2–3-linked sialic acid [Neu5Acα2-3Gal-β(1–3)-GalNAc]. Lectin staining showed that the binding of MAA to pancreatic islets was increased following DANA administration, but no increase was observed with saline treatment (Fig. 6A,B).

### Role of sialidase isozyme Neu3 in insulin release

**Figure 7:**
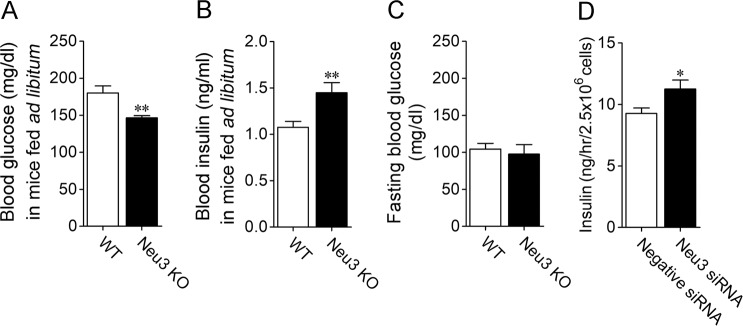
Regulation of insulin release by sialidase isozyme Neu3 in pancreatic islets. (**A**,**B**) Blood glucose and blood insulin levels following *ad libitum* feeding were measured in WT (*n* = 11) and Neu3 KO mice (*n* = 6). (**C**) Fasting blood glucose was performed in WT and Neu3 KO mice. *n* = 9. (**D**) Effect of Neu3 knockdown on insulin secretion induced by treatment of INS-1D cells with 8.3 mM glucose. *n* = 6. *P < 0.05 and **P < 0.01 (unpaired *t*-test).

Here, we investigated the contribution of Neu3 to insulin release. Under *ad libitum* feeding conditions, the blood glucose level in Neu3 KO mice was lower than it was in WT mice (Fig. 7A), while the blood insulin level in Neu3 knockout (KO) mice was higher than it was in wild-type (WT) mice (Fig. 7B). Fasting blood glucose levels measured after 24-hour fasting were not significantly different between WT and Neu3 KO mice (Fig. 7C). We also investigated the effect of Neu3 knockdown on insulin secretion. The insulin secretion by INS-1D cells that was induced by 8.3 mM glucose was further enhanced by treatment with a Neu3 siRNA (Fig. 7D).

## Discussion

A previous study showed that sialidase isozyme Neu1 mRNA is expressed abundantly in the pancreas^12^; however, the distribution and roles of sialidase in the pancreas are poorly understood. On the basis of our finding that pancreatic islets in the mouse pancreas exhibit intense sialidase activity, we examined the effect of the sialidase inhibitor DANA on insulin secretion. Insulin release from INS-1D cells induced by stimulation with 8.3 mM glucose was enhanced by the sialidase inhibitor DANA. In the mouse IPGTT, intravenous injection of DANA caused an increase in serum insulin levels that was enhanced to the same degree as that caused by a high dose of glibenclamide. These results suggested that DANA is a potentiator of glucose-induced insulin secretion. DANA (700 μmol/kg) significantly improves glucose tolerance without affecting ITT, implicating that DANA improves glucose tolerance by promoting insulin secretion.

Under non-stimulated conditions, DANA did not cause insulin release from INS-1D cells without cytotoxicity. Under fasting conditions, serum levels of insulin and glucose were not affected by intravenous injection of DANA. Hypoglycaemia is the most common and serious side effect of glucose-lowering drugs including sulfonylureas. Two gut incretin hormones, glucose-dependent insulinotropic polypeptide (GIP) and glucagon-like peptide-1 (GLP-1), enhance insulin secretion at only high blood glucose concentrations^8,13^. Since their effects are generated in a blood glucose level-dependent manner, the insulinotropic activity of incretin-based drugs exhibits antidiabetic effects with a low risk of hypoglycaemia. DANA is also expected to be useful as a glucose-dependent insulin secretagogue that has a low risk of hypoglycaemia.

Oral glucose administration induces much greater insulin release than intravenous or intraperitoneal glucose administration because secretion of incretin hormones is enhanced in response to ingestion of food or glucose in the small intestine^14–16^. On the other hand, GLP-1 levels were not increased but were transiently decreased, as shown by the IPGTT, while plasma insulin and glucose levels were found to be increased by the IPGTT^17^. Since insulin release induced by the IPGTT was enhanced by DANA, the insulinotropic effect of DANA seems to be induced without the release of incretin hormones.

Incretin hormones activate G protein-coupled receptors on β-cells, resulting in the facilitation of insulin exocytosis by raising the levels of intracellular cyclic adenosine monophosphate and [Ca^2+^]_i_^18–21^. We found that [Ca^2+^]_i_ oscillations induced by 8.3 mM glucose in INS-1D cells were not affected by DANA, although DANA enhanced insulin exocytosis. Enhancement of insulin secretion by DANA may thus be caused by increasing the efficiency of exocytosis without affecting Ca^2+^ signalling, which is a different mechanism from that of the action of incretin hormones. Expression of α2-3-linked sialic acid was detected by lectin staining at 30 min after glucose administration and was found to be increased in pancreatic islets following intravenous injection of DANA. Gangliosides and glycosphingolipids often containing α2-3-linked sialic acid, are involved in various plasma membrane functions. Recently, it was reported that gangliosides are continually and dynamically exchanged between raft domains and the bulk domain, which affects the membrane properties^22^. Sialic acid is essential for the membrane fusion process^23^. DANA may enhance insulin release by increasing sialic acid expression levels and by affecting the membrane fusion process.

The expression level of Neu3 mRNA in mouse pancreatic islets was remarkably higher than it was in exocrine tissue. Neu3 preferentially hydrolyses gangliosides and is involved in insulin signalling and insulin-resistant diabetes mellitus. DANA enhanced glutamate release in hippocampal neurons by increasing the content of ganglioside GQ1b/GT1a^6^. In this study, we investigated the contribution of Neu3 to insulin release using Neu3 KO mice. The Neu3 KO mouse line that we used inactivates the Neu3 gene in the whole body without affecting the mRNA levels of Neu1, Neu2 and Neu4; further, the mouse line exhibits decreases sialidase activity towards the ganglioside GM3, but there are no obvious abnormalities in the appearance and lifespan of the mouse^24^. Under *ad libitum* feeding conditions, the blood insulin level in Neu3 KO mice was significantly higher than it was in WT mice. The insulin secretion induced by 8.3 mM glucose treatment was also enhanced by Neu3 knockdown in INS-1D cells. These results suggested that inhibition of Neu3 activity contributed to the enhancement of insulin release and that Neu3 downregulates insulin release. Since blood glucose levels were not significantly different in WT and Neu3 KO mice under fasting conditions, Neu3 is thought to regulate insulin release depending on the blood glucose level.

Chronic overexpression of Neu3 results in the development of insulin resistance by reduction of insulin-stimulated phosphorylation of the insulin receptor and insulin receptor substrate I (IRS1) in skeletal muscle^5^. Neu3 sialidase activity induced by olanzapine, an antipsychotic agent associated with insulin resistance, attenuated insulin-induced phosphorylation of insulin growth factor receptor and IRS1, contributing to insulin resistance^25^. Hyperglycaemia induced by chronic intravenous injection of elastin-derived peptides was mitigated by DANA through the improvement of insulin sensitivity^26^. These findings support the idea that chronic inhibition of Neu3 with a sialidase inhibitor may improve insulin resistance under the condition of a high blood glucose concentration as well as insulin release.

On the other hand, transient overexpression of Neu3 in mouse livers using adenoviral vectors improved insulin sensitivity and glucose tolerance in mice^4^. A single injection of DANA did not affect insulin resistance^26^. In addition, the ganglioside GM3 induces insulin resistance^27^. Although Neu3 is a key enzyme involved in ganglioside hydrolysis^28–30^, GM3 content is hardly altered by treatment with an siRNA targeting Neu3^31,32^. However, it has been reported that Neu3 silencing results in GM3 accumulation^33^. Thus, it is necessary to closely examine the influence of DANA on insulin resistance.

There was no difference between Neu1-deficient and wild-type mice in circulating blood insulin levels after overnight fasting or at 30 min after intraperitoneal glucose injection^2^. These results suggest that Neu1 does not affect insulin secretion. Fougerat *et al*. reported that desialylation of the insulin receptor by Neu1 induces active conformation of the insulin receptor dimer^34^. Activation of the insulin receptor by insulin was attenuated by inhibition of endogenous Neu1 by pretreatment with 1 mM DANA. Although DANA inhibits all mammalian sialidase isozymes^35,36^, sialidase isozyme-selective inhibitors have recently been developed for Neu1, Neu2, Neu3 and Neu4^36–40^. Sialidase isozyme-selective inhibitors that do not inhibit Neu1 activity may thus be suitable for the treatment of diabetes mellitus.

Some limitations exist in this study. Transgenic mice in which Neu3 is forcibly expressed have been reported to exhibit different degrees of insulin sensitivity, which is tissue specific, as described above^4,5^. However, we used only Neu3 whole body knockout mice. It is possible that the blood glucose levels of our Neu3-deficient mice are affected by changes in insulin sensitivity as well as by potentiation of insulin secretion. Additionally, we used only healthy male mice to study the effect of DANA on insulin secretion. To clarify the effectiveness of DANA for the treatment of type 2 diabetes mellitus, verification using animal models of diabetes is required.

In conclusion, pancreatic islets in the mouse pancreas show intense sialidase activity. DANA enhanced insulin release induced by a high concentration of glucose, resulting in a decrease in blood glucose levels. Enhancement of insulin release by DANA, however, was not induced under fasting conditions. Therefore, DANA is a glucose-dependent potentiator of insulin secretion.

## Methods

### Animals

Male C57BL/6J mice (8–10 weeks old) were purchased from Japan SLC (Hamamatsu, Japan). Neu3 KO (*Neu3*^−/−^) mice in C57BL/6J genetic backgrounds were generated by the method described previously^24^. Briefly, heterozygous mice were bred and crossed to obtain littermates of WT and KO mice. Genotypes were determined by polymerase chain reaction (PCR) with DNA polymerase (KOD FX Neo, Toyobo, Japan) and primers (#1, GCTCTACCCCATTCTACATCTCCAGAC; #2, TCGTGCTTTACGGTATCGCCGCTCCCGATT and #3, GTGAGTTCAAGAGCCATGTTGCTGATGGTG).

The mice were housed under standard laboratory conditions (23 ± 1 °C, 55 ± 5% humidity) and had access to tap water and diet *ad libitum*. The lights were automatically turned on at 8:00 and turned off at 20:00. All experiments were performed in accordance with the guidelines of University of Shizuoka for the care and use of laboratory animals. The protocols were pre-approved by the animal ethical committee of University of Shizuoka.

### Sialidase activity imaging

BTP3-Neu5Ac was synthesized according to the procedure described previously^9^. Next, sialidase activity imaging was performed according to the procedure described previously^7,9^. Briefly, tails of pancreas were quickly harvested from C57BL/6J mice and then embedded in Tissue-Tek OCT compound (Sakura Finetechnical, Tokyo, Japan). After being frozen, the pancreas sections were cut into 100-μm-thick sections at −20 °C using a cryotome. The sections were stained with 1 mM BTP3-Neu5Ac in phosphate buffered saline (PBS) at 27 °C for 60 min. After washing with PBS, fluorescence was observed using a fluorescence microscope (IX71, Olympus) by using a filter set (ex/em, BP330-385/BA510IF). The background level of fluorescence was determined using a non-stained pancreas slice. In all observations with the fluorescence microscope, the gain of the DP70 Digital Microscope Camera (Olympus) was set in order to not detect background fluorescence. After obtaining pictures, the slices were fixed with 4% paraformaldehyde in PBS, then stained with hematoxylin-eosin.

### Measurements of insulin secretion

INS-1D cells, a pancreatic β-cell line, kindly gifted by Dr. C. Wollheim (University Medical Center, Geneva, Switzerland) were cultured for 48 hours in RPMI 1640 medium supplemented with 10% fetal bovine serum, 100 μg/ml streptomycin, and 100 U/ml penicillin, and then cells were preincubated in HEPES-buffered Krebs solution (HK solution: 129 mM NaCl, 4.75 mM KCl, 1.2 mM MgSO_4_, 2.54 mM CaCl_2_, 1.18 mM KH_2_PO_4_, 10 mM HEPES, 5 mM NaHCO_3_, 0.1% bovine serum albumin, 2.8 mM glucose; pH 7.4) for 60 min at 37 °C, and then incubated in HK solution containing 0–10 μM DANA or in HK solution containing 8.3 mM glucose and 0–10 μM DANA for 60 min. At the end of the incubation, 1 ml of the incubation medium was collected, and then centrifuged at 800 × g for 10 min. Insulin level in the supernatant was measured by using an ultra-sensitive mouse insulin enzyme-linked immunosorbent assay (ELISA) kit (Morinaga Institute of Biological Science, Japan).

### Measurements of sialidase activity

Sialidase activity was measured according to the procedure described previously^7,41^. Briefly, INS-1D cells or C6 rat glioma cells stably expressing the rat sialidase isozyme Neu3 were lysed in n-octyl-*β*-_D_-glucoside buffer. For measurements of sialidase activity, each lysate was incubated in 10 mM acetate buffer (pH 4.6) containing 40 μM 4MU-Neu5Ac at 37 °C for 60 min. After adding 500 mM of sodium acetate buffer (pH 10.7), the released 4-methylumbelliferone (4MU) was measured using a microplate reader (ex/em, 355/460 nm; Infinite M200, Tecan, Männedorf, Switzerland).

### Measurements of cytotoxicity

INS-1D cells were exposed to 10^−1^–10^5^ μM DANA. Released LDH was measured using a coupled enzymatic assay kit (CytoTox-OneTM, Promega, WI, USA).

### Intraperitoneal glucose tolerance test (IPGTT)

The IPGTT was performed using C57BL/6J mice after 24-hour fasting. DANA (0–2,100 μmol/kg b.w.) or glibenclamide (10 mg/kg b.w.) in saline was administered by intravenous or intraperitoneal injection, respectively. Thirty seconds later, glucose (2 g/kg b.w.) was administered by intraperitoneal injection. Glucose was measured from a tail venesection using a blood glucose meter (ACCU-CHEK ST Meter, Roche DC Japan, Tokyo, Japan) before administration of DANA or glibenclamide (0 min) and at 30, 60, 90 and 120 min after glucose administration. Blood was sampled at 120 min after glucose administration, and then plasma insulin levels were measured using an ELISA kit.

To investigate the effect of DANA on insulin secretion under the fasting condition, DANA (0–700 μmol/kg b.w.) and glucose (2 g/kg b.w.) were administered by intravenous injection and intraperitoneal injection, respectively, after 24-hour fasting. Blood was sampled before (0 min) and at 30 min after administration. Blood glucose and plasma insulin levels were measured as described above.

In the case of Neu3 KO mice, blood glucose and insulin levels were measured at 15:00 under the *ad libitum*-fed condition. Fasting blood glucose was also measured after 24-hour fasting.

### Insulin tolerance test

An insulin tolerance test was performed after 5-hour fasting. DANA (0–700 μmol/kg b.w.) was administered intravenously, and then insulin (2 mU/g b.w.; Humulin R, Eli Lilly Japan) was administered intraperitoneally 30 seconds later. Blood was sampled before administration of DANA (0 min) and at 30 min after insulin administration. Blood glucose and plasma insulin levels were measured as described above.

### Measurements of [Ca^2+^]_i_

INS-1D cells were loaded with 4 μM Fura PE-3/AM for 2.5 h at 37 °C in a chamber. Then the cells were continuously superfused with HK solution at a flow rate of 1 mL/min and observed by an inverted fluorescence microscope (IX71, Olympus, Tokyo, Japan). [Ca^2+^]_i_ was measured by dual-wavelength fluorometry (alternating excitation, 340 and 380 nm; emission, 510 nm) with an Aquacosmos/Ratio system (Hamamatsu Photonics, Hamamatsu, Japan). The 340/380 ratio fluorescence images indicating relative [Ca^2+^]_i_ were captured every 10 s. Glucose (8.3 mM) and/or DANA (10 μM) were applied in the superfusing HK solution. INS-1D cells showing increases in [Ca^2+^]_i_ by responding to 0.3 mM tolbutamide were identified as healthy β-cells^42^.

### Lectin staining

After 24-hour fasting, saline or saline containing DANA (2,100 μmol/kg b.w.) was administered to C57BL/6J mice. At 30 min after glucose administration, tails of pancreas were quickly harvested and embedded in Tissue-Tek OCT compound. After being frozen, the pancreas sections were cut into 20-μm-thick sections at −20 °C using a cryotome. After fixation with 4% paraformaldehyde in PBS, blocking was performed in Carbo-Free Blocking Solution (Vector Laboratories, Burlingame, CA). The sections were stained with FITC-labelled MAA (0.1 mg/ml, EY Laboratories, San Mateo, CA) and DAPI (1 µg/ml DAPI). Images were acquired using a fluorescence microscope (BZ-X710, Keyence, Osaka, Japan) and filter sets (ex/em: 450–490 nm/500–550 nm for FITC and 340–380 nm/above 400 nm for DAPI). Fluorescence intensity was measured by using Photoshop CS4 (Adobe Systems, San Jose, CA). The background level of fluorescence was determined by using non-stained sections.

### Real-time quantitative reverse transcription-PCR (real-time RT-PCR)

Pancreatic islets and exocrine tissues were isolated from mice by the collagenase digestion technique as described previously^43^. Total RNAs were isolated from pancreatic islets and exocrine tissues using an RNeasy Mini kit (Qiagen, Hilden, Germany) according to the manufacturer’s instructions. Copies of cDNA were evaluated using a thermal cycler system (Thermal Cycler Dice® Real Time System Lite, TaKaRa Bio), a One Step SYBR PrimeScript PLUS RT-PCR kit (Perfect Real Time, TaKaRa Bio) and primer pairs (5-AGGGCAGGGGTTCATGGT-3′ and 5′-GATGAAGGGGCGCTCTGTA-3′ for Neu3, and 5′-CCATTTTGTCTACGGGACGA-3′ and 5′-AAGGCAAAAGACACCGTCAAG-3′ for glyceraldehyde-3-phosphate dehydrogenase). To normalize for sample variation, GAPDH mRNA was used as an internal standard.

### RNA interference experiments

siRNA against Neu3 was purchased from Thermo Fisher Scientific (Stealth siRNA no. RSS300799; Waltham, MA). An equivalent amount of scrambled siRNA (AllStars Negative Control siRNA; Qiagen, Hilden, Germany) was used as a negative control. INS-1D cells were transfected with siRNA by electroporation (CLB-Transfection devices; Lonza, Basel, Switzerland) according to the manufacturer’s protocols.

### Statistical analysis

Statistical significance was assessed by the unpaired *t*-test or by ANOVA with Bonferroni’s multiple comparison test or Dunnett’s multiple comparison test. The nonparametric Kruskal-Wallis test with Dunn’s multiple comparison test or unpaired *t*-test with Welch’s correction was performed when data had different variances. Statistical analysis was performed by GraphPad prism (GraphPad Software, La Jolla, CA). Error bars are expressed as standard errors of the mean.
